# Data-Driven Health Monitoring of Construction Materials Based on Time Series Analysis of Crack Propagation Sensors

**DOI:** 10.3390/ma19071317

**Published:** 2026-03-26

**Authors:** Paulina Kurnyta-Mazurek, Artur Kurnyta

**Affiliations:** 1 Faculty of Mechatronics, Armament and Aerospace, Military University of Technology, 00-908 Warsaw, Poland; 2 Airworthiness Division, Air Force Institute of Technology, 01-494 Warsaw, Poland; 3 Łukasiewicz Research Network—Institute of Aviation, 02-256 Warsaw, Poland

**Keywords:** time series model, ARIMA model, customized crack propagation sensor

## Abstract

The paper investigates the applicability of time series models for processing data obtained from a customized crack-propagation sensor. Because the sensor records a variable and noise-affected waveform, the study focuses on models capable of forecasting signals composed of both trend and stochastic components. Adaptive, analytical, and autoregressive approaches were examined, with particular attention to their suitability for short, non-stationary sequences typical of fatigue-related measurements. Based on the statistical characteristics of the sensor output during crack growth, the ARIMA model was selected for further analysis and algorithm development. The forecasting performance of ARIMA was evaluated for different parameter configurations by comparing the range and variability of the base and predicted data. Initial tests using first-order parameters produced unsatisfactory results, with high variance observed in both raw and modeled signals. Therefore, model parameters were optimized using the *aicbic* function, and the analyses were repeated. For the selected datasets, variance reduction by 3–4 orders of magnitude was achieved, demonstrating a substantial improvement in prediction stability. The presented results confirm that the proposed methodology is effective for processing complex sensor signals and highlight the broader significance of applying statistically grounded time series models in structural health monitoring. The study introduces an innovative framework for evaluating fatigue-related sensor data and establishes a reliable baseline for future predictive methods.

## 1. Introduction

Time series modeling and forecasting play a crucial role in various technical applications. In this field, a time series means a sequence of significant values recorded at discrete time intervals that provide a basis for predicting future values. Forecasting accuracy depends on the chosen model and the prediction horizon. The pertinence typically tends to decrease as the horizon is extended. Selecting an appropriate model is essential to ensure optimal accuracy, regardless of the prediction horizon [1].

Analysis of time series is of great importance in finance, economics and logistics. In finance it is used to forecast, e.g., stock prices, interest or exchange rates, in economics to analyze patterns, and in logistics and inventory management to predict demand for goods. The paper [2] provides an example of time series prediction of demand forecasting in the regular passenger transport industry. Additionally, time series analysis examples in the area of finance can be found in [3,4,5]. In the paper [3], the analysis and prognosis algorithm based on fuzzy clustering was presented. In turn, work [4] shows hybrid approach in investment strategies, where the LSTM-ARIMA (Long Short Term Memory-Autoregressive Integrated Moving Average) model was used to realize both nonlinear patterns and linear relationship. Moreover, paper [5] again presents hybrid time series model to predict exchange rates. In this case, ARIMA-SARIMA and LSTM models were used.

Time series are also widely utilized in sales analysis, production planning, weather forecasting and ecology, medical research, bioengineering, business process monitoring and optimization. Analysis of the spatial characteristics of local precipitation was introduced in [6]. The predicted results obtained with use of the ARIMA time series model were compared with the measured values. Obtained observations showed a high degree of consistency. Moreover, paper [7] shows a forecasting algorithm of air temperature and rainfall in Mymensingh (Bangladesh) for the purpose of aquaculture studies. The impact of various water quality parameters and fish habitat conditions were examined. In the field of ecology, papers [8,9] present time series analysis implementation for prediction of air quality. The univariate and multivariate time series models were used in [8] to perform analysis. It indicated that the forecasting algorithm using the ARIMA model produced more accurate results than the VAR (Vector AutoRegression) model. Additionally, paper [9] shows forecasts of global land-based ocean temperature with the use of an ARIMA time series model as well. Moreover, papers [10,11,12] present time series modeling implementation in bioengineering. In [10], time series analysis was used to detect the gait cycle from a foot pressure sensor attached to the heel and thenar. Meanwhile, multidimensional time series was applied to protein structure classification in [11]. Additionally, with the use of ARIMA and SARIMA time series forecasting, hive weight and in-hive temperature were analyzed in [12].

Examples of forecasting with the time series models can also be found in technical engineering, e.g., for monitoring and control systems [13] or to predict electric energy consumption [14,15]. Paper [14] presents building energy consumption, which is a non-stationary and nonlinear process. For that reason, a hybrid algorithm based on Support Vector Regression (SVR) and Empirical Mode Decomposition EMD was used. Moreover, the prediction of energy consumption with the use of a modified ARIMA model was presented in [15]. Combined Random Forest (RF) ARIMA model with the advanced feature selection, Minimum Redundancy Maximum Relevance and Maximum Synergy MRMRMS method was applied to produce a sparse model. The approach used was 40% more effective than the second-best alternative. The use of time series for energy consumption analysis is of great importance for optimizing production capacity, improving efficiency, and effectively managing energy resources.

Furthermore, the time series analysis was widely used in materials research [16,17,18]. In [16], three temporal prediction models, namely Artificial Neural Network (ANN), Random Forest (RF), and Long Short Term Memory (LSTM) algorithm, were used for creep forecasting of low-carbon concrete materials. In [17], time series models were found effective in prognosis of carbon steel corrosion progression in an extreme acid environment. Finally, in [18], the ARIMA model was used to predict erosion-induced damage.

Big data analysis is another field for application of time series modeling [19,20,21,22]. In [19], results of a projected and implemented in distributed system for processing and analyzing time series data on a large scale, based on the Spark platform, were presented. Moreover, time series analysis and decomposing algorithm into long-term and short-term change trends were shown in [20]. Furthermore, trends prognosis with the use of the Holt-Winters model was presented in [21] and ARIMA-based hybrid model were applied in the field of edge computing in [22].

The ARIMA model and its modifications are widely used in technical issues requiring the forecast of future values. This is why it was chosen as a data processing algorithm from crack propagation sensor, delivered within this work.

The content of this paper is organized as follows: the first section introduces the time series models; following this, mathematical models of adaptive, analytical and autoregressive models are described. The third section presents results obtained from fatigue crack growth investigation with use of the Customized Crack Propagation Sensors (CCPS) as well as the ARIMA-based predictive algorithm. After this, several experimental results are presented, along with an analysis. It should be remembered that these measurement findings present the most important validation results of the crack propagation studies. Finally, some concluding remarks have been formulated, which reflect the effects of the studies carried out in this paper.

The contribution of this work enhances and verifies the analytical solutions for time series data, acquired by a sensor network, which is devoted to structural health monitoring of the host structure. The methodology is delivered for utilization and adaptation of ARIMA model, with sufficient evaluation of its performance for crack development monitoring with CCPS. Time series datasets from several sensors were analyzed, to avoid misleading conclusion of adopted method effectiveness for different crack grow rates in the specimen.

## 2. Materials and Methods—Time Series Models

**Figure 1:**
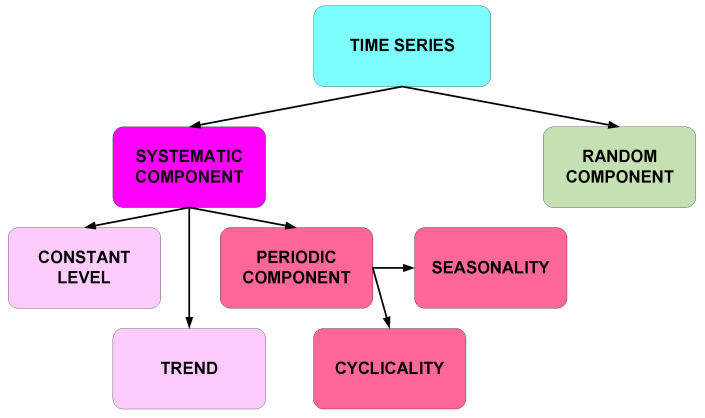
Components of time series model.

The analysis of a time series begins with determining its type, by exploration of signal components. In most cases, any time series will be characterized by the presence of two basic components: a deterministic and a random component. The deterministic component can be characterized by a constant value of the variable, locally monotonic trend, periodically occurring seasonal or cyclical fluctuations. The periodic component can occur together with a constant level of the value of the variable or with a trend. Depending on the phenomenon or process under consideration, the influence of these components on the unraveling sequence of values will vary. The components of the time series are shown in Figure 1.

In the following part of the article, adaptive, analytical, autoregressive and other models are described in detail.

### 2.1. Adaptive Models

Adaptive time series models are used when the model and its parameters change while forecasting the value of a numerical sequence. This group of prediction methods includes naive methods and time series smoothing algorithms, among others. Adaptive methods of data analysis are usually used for short-term forecasts.

Naive methods are also best suited for short-term forecasting. Their main advantage is uncomplicated computational implementation. Naive methods can be used for series with a constant level of the variable, and for series with a linear and exponential trend, including the phenomenon of seasonality. The last method will not be analyzed in the study, since the sequence of numerical values recorded by the crack detection sensor acquisition system does not show the characteristics of seasonality or periodicity of the data.

To eliminate random fluctuations of the time series, smoothing algorithms are usually used, which allow us to make predictions of the value of the sequence, usually with a displacement of one moment of time. These algorithms are divided into two groups: those using moving average technique and exponential smoothing. Moving average methods allow you to make a prediction one time step ahead.

For time series in which there is no trend, the simple or weighted moving average method is used. The use of the first method involves using the following relationship:

(1) $${\overset{ˇ}{y}}_{t+1}=\frac{1}{k}\overset{t}{\underset{i=t-k+1}{\sum }}y_{i},$$

where *k* is the smoothing constant. The next values of the smoothed data sequence are the arithmetic averages of *k* previous observations. The determination of the constant k involves calculating one of the average errors of the smoothed predictions and choosing such a value of the constant for which the smallest error is obtained.

The weighted moving average method, on the other hand, uses the following equation:

(2) $${\overset{ˇ}{y}}_{t+1}=\overset{t}{\underset{i=t-k+1}{\sum }}{w_{i-t+k}\cdot y}_{i},$$

where *w_i_* are the weights set by the forecaster, satisfying the relation:

(3) $$\overset{k}{\underset{i=1}{\sum }}w_{i}=1.$$

By determining the value of successive weights, we indicate the validity of individual past elements of the numerical sequence.

The second group of algorithms for smoothing a time series are exponential methods. These primarily include the Brownian, Holt and Winters methods.

### 2.2. Analytical Models

Mathematical relationships are used when applying analytical methods to predict time series data. With these methods, it is possible to extrapolate future values of a numerical sequence further than just one time step forward. Determining the prediction involves calculating the value of the appropriate formula for a selected future moment in time. In this approach (passive method), the invariability of the adopted model is assumed.

Analytical methods can be used to forecast the value of a time series on the basis of a trend function or seasonal fluctuations. Again, due to the nature of the data registered by the crack detection sensor, only the first group of methods will be considered in the study.

In the first stage of the process of developing a forecast based on the trend function, it is assumed that there are only random fluctuations in the time series in addition to it. Then the choice of the form of the model of the series (additive or multiplicative model) is made and its parameters are determined. Then the verification of the prepared model is carried out, the determination of forecasts and their evaluation is performed.

The most commonly used model of the trend function is a linear relationship [23]:

(4) $$f_{t}=\beta_{0}+\beta_{1}t,$$

where consecutive moments of time are indexed with the numbers *t* = 1, 2, …, *n*. Based on it, we get a model of the time series in the form of an equation:

(5) $$y_{t}=\beta_{0}+\beta_{1}t+e_{t}.$$

Further estimation of the model parameters is carried out using the relationship in matrix form:

(6) $$Y=X\hat{\beta }+e$$

or after conversion

(7) $$e=Y-X\hat{\beta },$$

where the matrices take the following form:

(8) $$Y=\left[\begin{array}{c} y_{1}\\ y_{2}\\ \begin{array}{c} \vdots \\ y_{n} \end{array} \end{array}\right],\;X=\left[\begin{array}{cc} 1 & 1\\ 1 & 2\\ \begin{array}{c} \vdots \\ 1 \end{array} & \begin{array}{c} \vdots \\ n \end{array} \end{array}\right],\;\hat{\beta }=\left[\begin{array}{c} {\hat{\beta }}_{1}\\ {\hat{\beta }}_{2} \end{array}\right],\;e=\left[\begin{array}{c} e_{1}\\ e_{2}\\ \begin{array}{c} \vdots \\ e_{n} \end{array} \end{array}\right].$$

The least squares method is usually used to determine the parameters of the model. Based on it, we obtain an estimate of the time series value in the form of equation [23]:

(9) $${\hat{y}}_{t}={\hat{\beta }}_{0}+{\hat{\beta }}_{1}t.$$

After determining the parameters of the time series model, it is verified by comparing the data determined from the model and the data obtained experimentally.

### 2.3. Autoregressive Models

When using autoregressive models, it is assumed that there is a relationship between the values of a time series variable at moments away from each other. In other words, it is assumed that based on information about the past and current values of a numerical series, we can forecast its values in the future. Autoregressive models can be used only for time series that are stationary or that can be reduced to a stationary form.

The most widely used autoregressive model used in monitoring and control systems to forecast future values of one-dimensional time series is the ARIMA model, also known as the Box–Jenkins model. It consists of three components: the autoregressive process AR, the moving averages MA, and the degree of integration I.

The autoregressive component AR(*p*) uses the process memory and estimates the current value as a weighted linear sum of the previous values of the time series [24]. The AR component is characterized by the order labeled *p*, which determines how many previous values affect the current value of the process. The AR component is described by the equation:

(10) $$y_{t}=\rho_{1}y_{t-1}+\rho_{2}y_{t-2}+\cdots +\rho_{p}y_{t-p}+\varepsilon_{t},\;\;for\;t=1,\;2,\;\ldots ,p$$

where *y_t_* is the value of the time series at time t (predicted value), and *y_t_*_−1_, *y*_*t*−2_, …, *y*_*t*−*p*_ are the values of the time series at times *t* − 1, *t* − 2, …, *t* − *p*. In turn, ε_t_ denotes the random component or disturbance at time *t*. The coefficients *ρ*_1_, *ρ*_2_, …, *ρ_p_* are the parameters of the AR process and indicate the effect of previous series values on the current value of the time series [25].

The MA(*q*) component that determines the moving average process is similar to the autoregressive process. In this case, the current value of the time series depends on the disturbances at time *t* and at times *t* − 1, *t* − 2, …, *t* − *q*. The parameter *q* is the order of the MA process, expressed by the equation:

(11) $$y_{t}=\beta_{1}\varepsilon_{t-1}+\beta_{2}\varepsilon_{t-2}+\cdots +\beta_{q}\varepsilon_{t-q}+\varepsilon_{t},\;\;dla\;t=1,\;2,\;\ldots ,q$$

where the coefficients *β*_1_, *β*_2_, …, *β_p_* are the parameters of the MA process and determine the effect of previous disturbance values on the present value of the time series.

The last component of the ARIMA model is integration I, characterized by the parameter *d*, which determines the degree of integration of the time series. This component of ARIMA makes it possible to bring the process described by the time series to a stationary form.

In summary, the ARIMA model has three parameters that must be defined during the modeling process: the order of the AR component—*p*, the order of the MA component—*q* and the degree of integration—*d*. In the next chapter, the model adaptation and evaluation is delivered, based on iterative change of its parameter due to additional, quantitative model performance analysis by autocorrelation and variance.

## 3. Results

As mentioned in the introduction, time series analysis is widely applied across various technological domains, including economics, automation, as well as monitoring and diagnostics. The applications of that analytical method delivered in this work are processing and prediction of the crack size detected by Customized Crack Propagation Sensor (CCPS). For that purpose, the ARIMA model was selected because the analyzed signals exhibit clear temporal dependencies, non-stationary behavior, and relatively short historical sequences, which makes classical machine learning or deep learning approaches less suitable. ARIMA offers several advantages in this context: it provides transparent parameterization, allows explicit control over trend and autocorrelation components, and performs reliably on short, noisy time series typical for sensor-based measurements.

### 3.1. Customized Crack Propagation Sensors and Laboratory Stand

**Figure 2:**
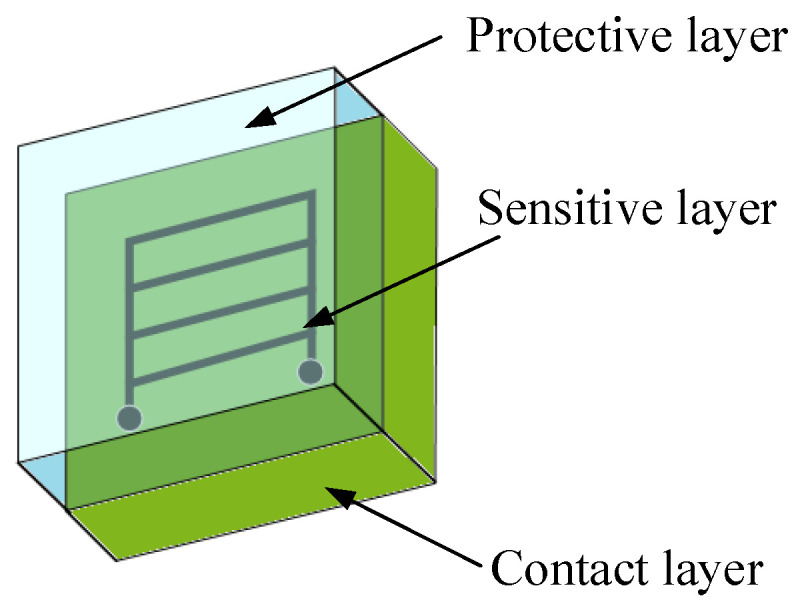
Layers of CCPS.

CCPS is composed of three layers, including a contact layer, a protective layer, and a sensitive layer [7], as shown in Figure 2. The first two layers were manufactured using polymeric material (MG Chemicals 832C epoxy resin, MG Chemicals, Burlington, ON, Canada). In contrast, the sensitive layer is created using electrically conductive paste, filled with silver flakes (Dycotec DM-SIP-3071S, Dycotec Materials Ltd., Wiltshire, UK). The properties and construction of the CCPS, its concept of operation and output signal description can be found in [13,26].

The principle of operation of this sensor is similar to parallel connection of resistors. The sensor needs to be bonded to the specimen or component, connected to the dedicated measurement system with signal conditioning and acquire its output voltage signal. When the crack propagates under the sensitive layer of the sensor, this causes the electro-conductive grid to break evenly with the host structure. Breaking a conductive fragment of the grid leads to an increase in the sensor equivalent resistance and a change in the measured voltage by the acquisition unit appears. Optical measurement of the sensor geometry and its distance from the notch after bonding enables rescaling the current sensor readings to the crack length estimation during damage propagation.

For sensors presented within this paper, the expected change in the measured signal after breakage of grid element is the same as for the parallel connection of even-valued resistors. Six-element sensitive grid CCPS were installed of the host specimen, so 6 transients of the voltage signal level were observed for each sensor.

Dedicated data acquisition systems were designed for this type of sensor, allowing measurements of the sensors’ voltage and informing about the development or absence of a crack. The system provides real-time information about the condition of the structure, enabling the continuous monitoring of its technical condition. Still, an appropriate data processing algorithm is necessary to analyze the results from CCPS. For this reason, the use of a dedicated algorithm will enable a proactive and predictive approach to damage monitoring with a potential to determine the time until the pre-failure state is reached.

**Figure 3:**
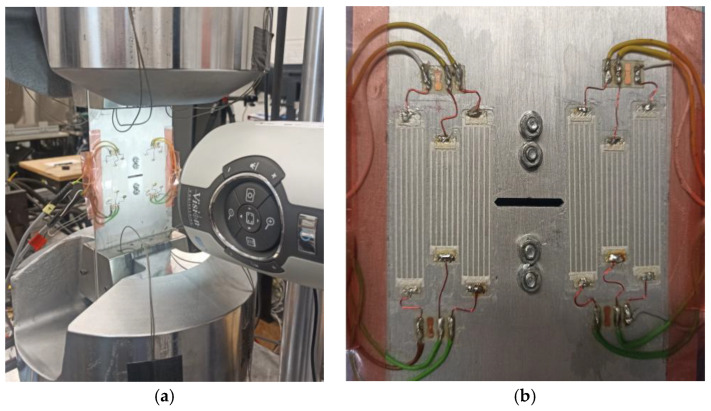
Test rig: (**a**) fatigue tests machine, (**b**) CCPSs mounted on the specimen.

To develop a data processing algorithm for predicting the rate of crack growth, example time courses recorded during fatigue test of a structure with CCPS were analyzed. Dedicated test rig is shown in Figure 3a, which is MTS 810.23 servo-hydraulic materials tester (MTS-Systems, Eden Prairie, MN, USA). The front side of the specimen with six CCPS mounted is presented in Figure 3b. The test rig induced the cyclic mechanical loading to the instrumented specimen, fixed in tester grips. The sinusoidal-type constant amplitude loading was used, with an amplitude of 11 kN and 0.5 cycle asymmetry ratio to develop fatigue crack in the specimen. The area where the crack propagation started was the central part of the sample, from the created notch, almost equally in both directions.

**Figure 4:**
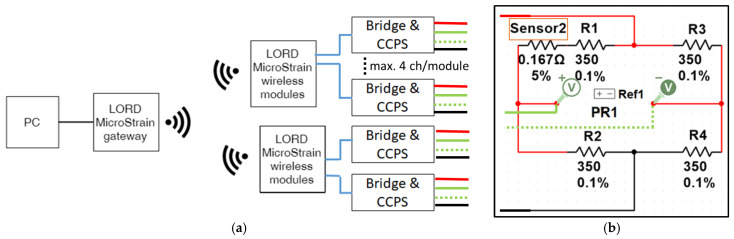
Scheme of the acquisition systems: (**a**) major scheme, (**b**) electric circuit of the sensors’ conditioning measurement bridge.

**Figure 5:**
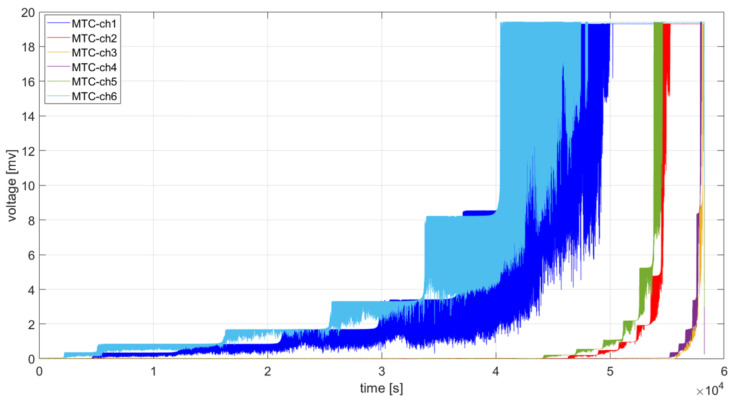
Examples of data recorded by the acquisition system of the CCPS.

Voltage signals from CCPS were measured by the dedicated data acquisition system. Its scheme and signal conditioning circuit to cooperate with the sensors is shown in Figure 4. Data acquisition system is composed of two V-Link 200 multichannel sensors modules (MicroStrain, Williston, VT, USA). A single V-Link-200 module provides four analog channels, compatible with a Wheatstone bridge measurement circuits using 120, 350, and 1 kΩ resistors with fixed 4.096 V bridge excitation. For each measuring channel, the sensor was fitted in series in one arm of a Wheatstone Bridge composed of 350 Ω precise resistors R1 ÷ R4 (Elpod, Cracow, Poland) (Figure 4b). Each CCPS was sampled with a frequency of 128 Hz. V-Link-200 provides a wireless connection with the PC via the gateway WSDA-200-USB (MicroStrain, Williston, VT, USA) to acquire data remotely from the specimen. During performed studies, data recorded by six sensors were analyzed. An example of registered data is shown in Figure 5.

In the case of the considered time courses shown in Figure 5, it is possible to distinguish a component with a constant level and a rising trend. The constant level of the variable is characterized by local maximum voltage level with oscillations caused by mechanical cycling of the specimen. The trend, on the other hand, is characterized by a tendency for the values of the components of the time series data to change in an ascending manner due to sensor grid element breakage. During the detailed analysis of the data, it will be necessary to determine the time intervals for which the constant component and the trend can be distinguished. At a further stage, appropriate time series models will be adopted and tests will be carried out on their compatibility with the estimated data.

### 3.2. Preliminary Tests of the Prediction Algorithm

The prediction of crack propagation was carried out using MATLAB 2024a software. A univariate ARIMA model was developed for the voltage time series using the *arima*(*p*,*d*,*q*) function [26,27]. During the modeling process, the parameters *p*, *d*, and *q* were defined as non-negative integers (1,1,1), as previously described. The developed algorithm also utilized MATLAB program *estimate* and *forecast* functions. The parameters of the applied algorithm are shown in Table 1.

Model fitting and time series prediction were performed iteratively. In each iteration, the algorithm forecasted 1000 future values of the series based on a dataset consisting of approximately 20,000 samples [26]. The orders of all ARIMA model parameters were assumed to be equal to 1.

**Figure 6:**
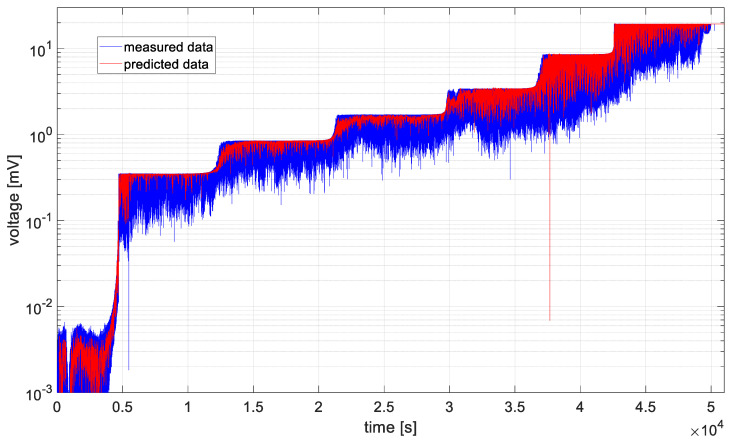
Data recorded by the acquisition system of the CCPS (MTC_ch1).

**Figure 7:**
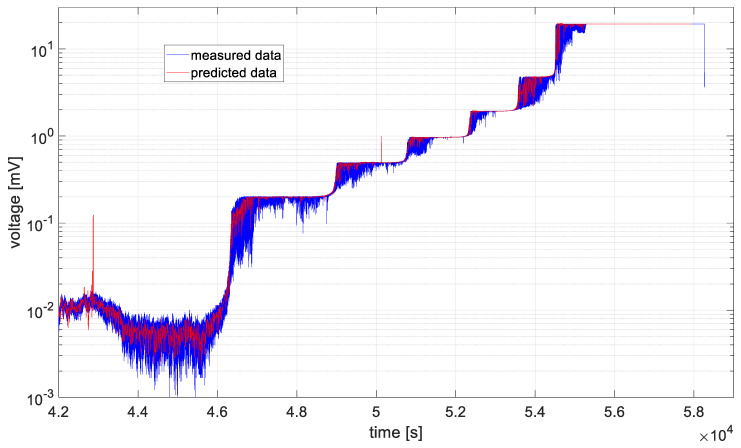
Data recorded by the acquisition system of the CCPS (MTC_ch2).

**Figure 8:**
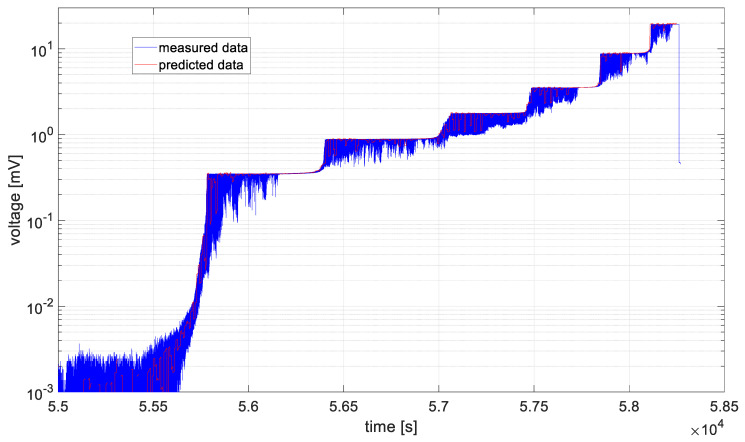
Data recorded by the acquisition system of the CCPS (MTC_ch3).

**Figure 9:**
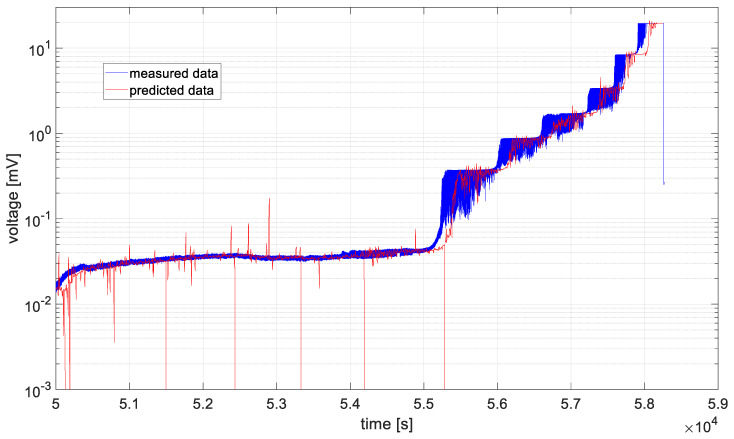
Data recorded by the acquisition system of the CCPS (MTC_ch4).

**Figure 10:**
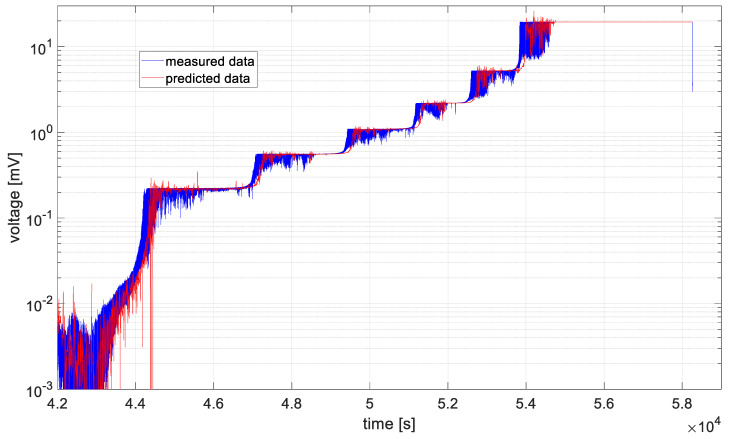
Data recorded by the acquisition system of the CCPS (MTC_ch5).

**Figure 11:**
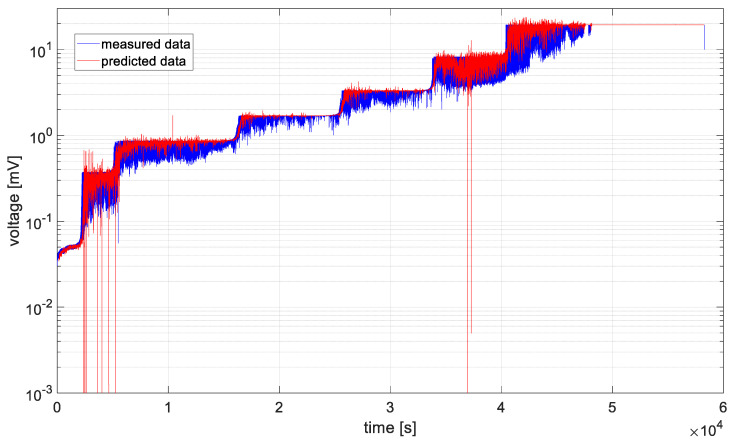
Data recorded by the acquisition system of the CCPS (MTC_ch6).

In Figure 6, Figure 7, Figure 8, Figure 9, Figure 10 and Figure 11, time series values recorded by data acquisition systems with CCPS and predicted values with the use of the ARIMA algorithm are presented. Measured and estimated data are marked with blue and red lines, respectively. Due to the nature of the time series data presented in Figure 7, Figure 8, Figure 9 and Figure 10, a close-up of the final hours of the fatigue test was created to visualize the accuracy of the algorithm’s performance.

**Figure 12:**
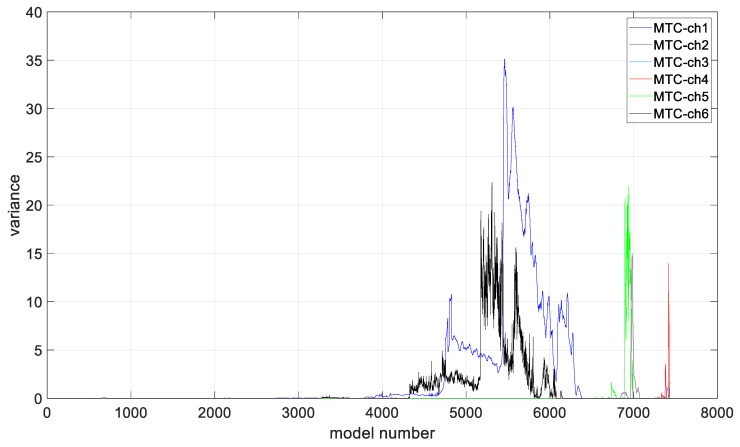
Variance values of the generated models in each of the analyzed channels.

In the preliminary tests, the simplest form of the ARIMA model parameters, equal to one, was assumed. Based on the presented figures, it is difficult to assess the effectiveness of the prepared algorithm; therefore, for each model in the set, the variance was calculated, and its values are shown in Figure 12.

### 3.3. Automatic Estimation of ARIMA Model Parameters

**Figure 13:**
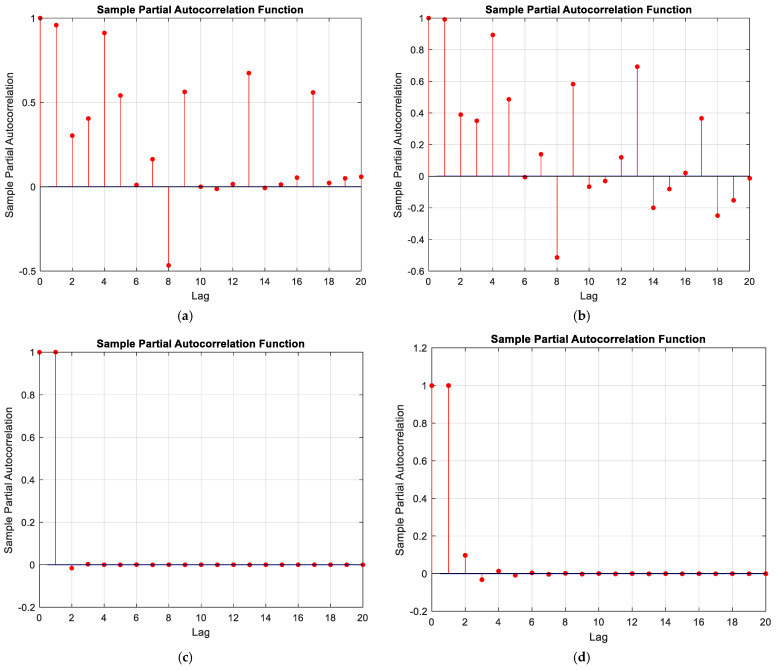
Partial autocorrelation function (PACF): (**a**) data from channel (MTC_ch1), (**b**) data from channel (MTC_ch4), (**c**) integrated data from channel (MTC_ch1), (**d**) integrated data from channel (MTC_ch4).

In the previous subsection, the prediction of crack propagation was performed using an ARIMA time series model with basic parameters set as (*p* = 1, *d* = 1, *q* = 1). The forecasted data consisted of 1000 consecutive samples, while the range of the base data was 20,000 samples. The ARIMA time series model can be applied to stationary datasets; therefore, the partial autocorrelation function (PACF) was examined for all measured data series. In all cases, a clear reduction in values was observed after a single integration of the time series. Figure 13 presents two examples of PACF plots before and after the integration process for sensors 1 and 4, which represented two different acquisition systems.

Based on the behavior of the autocorrelation function for successive lags, the effectiveness of applying a single integration can be observed. For all recorded data, setting the parameter *d* = 1 is sufficient to obtain a stationary series, as evidenced by autocorrelation values approximately equal to 1 and decreasing for subsequent lag values. However, slightly higher values can be observed for the second and third lag for the integrated signal of channel 4 (MTC_ch4).

After confirming that a single integration is sufficient to obtain a stationary series, an algorithm was designed to compute the optimal values of the parameters *p* and *q* for a given time series. For this purpose, the *aicbic* function from the MATLAB library was used. This function computes information criteria based on the log-likelihood values obtained by fitting competing models to the data. To select the best model, the criterion value must be calculated for each proposed model. This is a non-trivial task, as the number of models grows exponentially with respect to the number of variables. To test the correctness of the algorithm’s operation, it was assumed that models with a maximum autoregressive order *p* and moving average order *q* equal to 10 would be examined, for one channel from each data acquisition device. Therefore, the optimization was performed for *p* and *q* over the range 1–10 with a step size of 1, since both parameters are required to take integer values.

**Figure 14:**
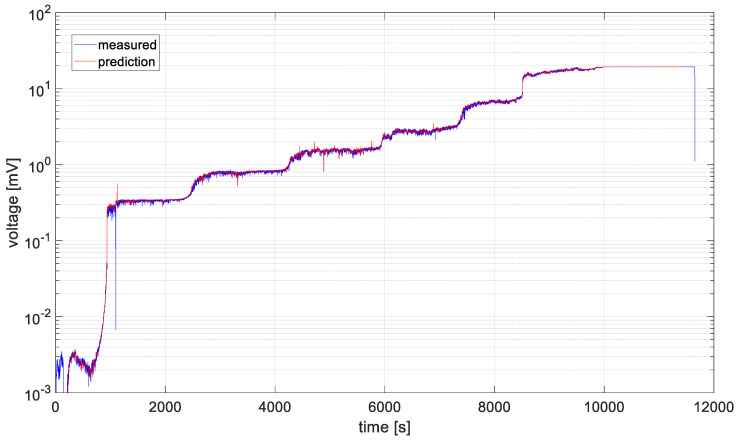
Averaged data from channel 1 subjected to prediction.

For the data recorded by the first acquisition channel, the algorithm computed the optimal values of *p* and *q* as seven and four, respectively. Unfortunately, the program intended to predict the voltage values from the sensor was unable to compute a stable form of the autoregressive polynomials for unprocessed time series. For this reason, it was decided to average the measured values and then repeat the optimization of the parameters *p* and *q*, which were found to be 5 and 8, respectively. The result of the algorithm’s operation for the averaged data values and the optimally selected ARIMA model parameters is shown in Figure 14.

**Figure 15:**
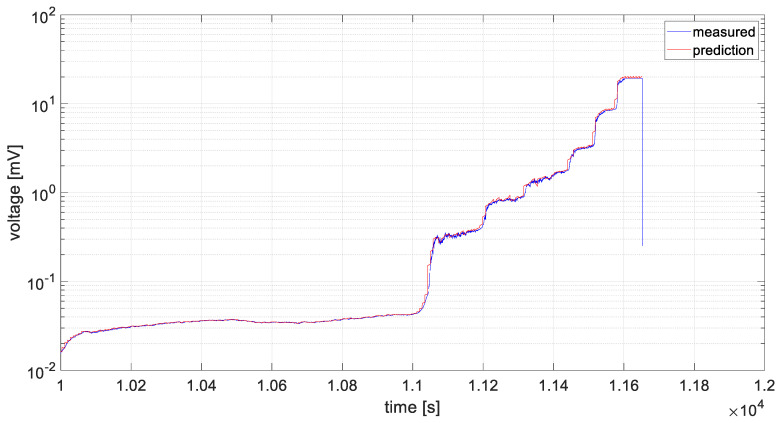
Averaged data from channel 4 subjected to prediction.

The same data processing scenario was applied to the fourth channel, representing the second acquisition module. The data were averaged and the ARIMA model parameters were optimized; in this case, both *p* and *q* were equal to 5. The effect of the prediction algorithm for the averaged data from the fourth channel is shown in Figure 15.

**Figure 16:**
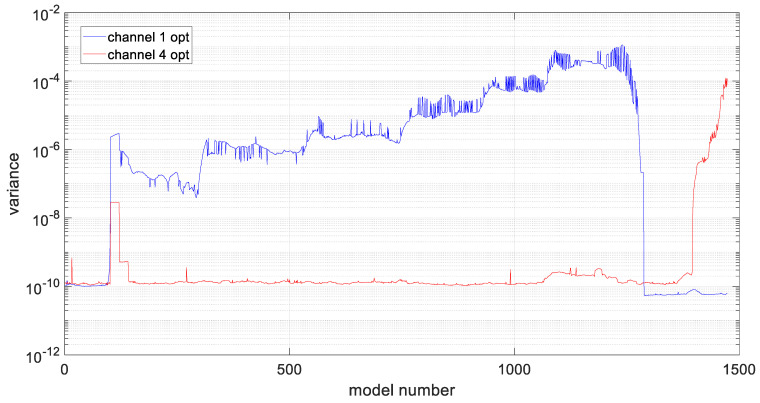
Variance values of the generated models in each of the analyzed channels.

**Figure 17:**
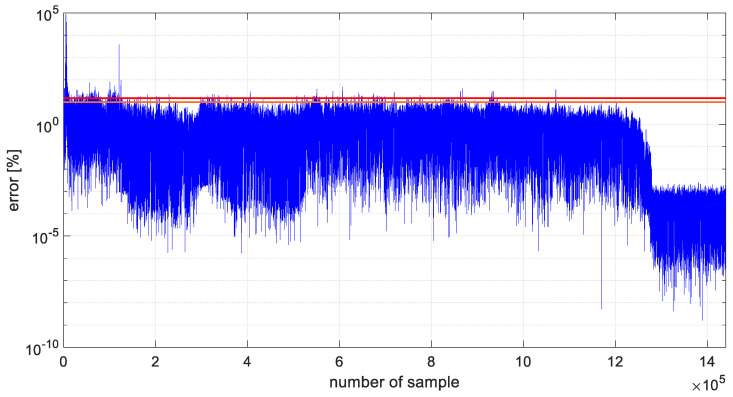
Prediction error calculated for channel 1.

**Figure 18:**
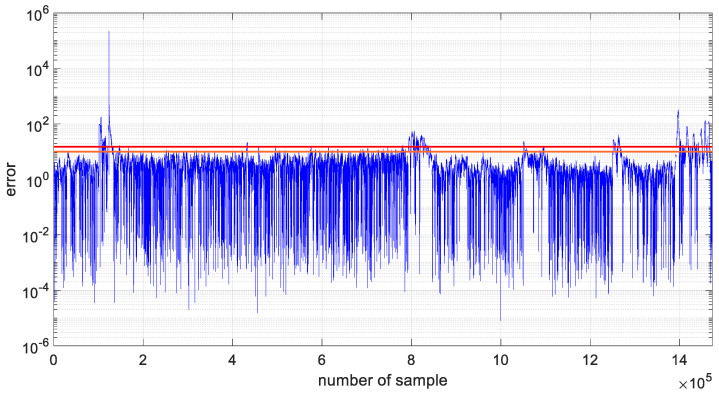
Prediction error calculated for channel 4.

As in the case of the preliminary studies of the developed algorithm, the variance values were recalculated for the two analyzed datasets, and these values are shown in Figure 16. Additionally, in Figure 17 and Figure 18, prediction errors calculated for both channels were presented.

## 4. Discussion

The presented results show that the size of the crack could be predicted with the use of ARIMA modeling. During the analysis, more than 7000 iterations were made for more than 7 million samples of time series voltage. In each iteration, the ARIMA model parameters were estimated, and 1000 future series values were predicted based on almost 20,000 samples. In the paper, we analyzed data from six CCPS mounted on one specimen. Calculation time and the number of generated models are shown in Table 2. The calculations were conducted using a computer with the following specifications: 13th Gen Intel Core i9 processor and 32 GB RAM.

Based on the table above, it can be observed that the shortest computation times are characteristic of cases 2 and 3. These cases are also characterized by the smallest signal level changes, as shown in Figure 7 and Figure 8. On the other hand, the longest computation times are associated with cases described by the largest fluctuations in the recorded voltage, i.e., cases 1 and 6, as shown in Figure 6 and Figure 11.

In all analyzed cases, the prediction algorithm for crack size with the ARIMA model proved effective in making forecasts. However, in cases 2 and 3, within the range of constant value progression, some impulsive changes in the predicted values can be observed. A temporary upward trend in the measured time series values or an error in the algorithm could be the cause. To improve accuracy, it would be advisable to test the algorithm for these cases with different series parameters, such as AR and MA equal to 2, or by increasing the integration degree of the series. Additionally, in the last case, the results shown in Figure 11, the largest overestimations of the series values occur. In this case, preliminary data processing could be attempted before proceeding with the prediction process. Moreover, the best fit between the predicted and measured data during the onset of the cracking process occurs in cases 2 and 3, where the smallest data fluctuations (resulting in significant data smoothing) were observed.

Figure 9, Figure 10 and Figure 11 show large time shifts between the measured and predicted data especially for acquisition channels 4–6. In the case of predicting crack propagation in channels 1–3, the estimate function had the parameter *Y*0 (presample response data) set to 2 (Mdl.P + 1). This is the number of presample observations required to initialize the AR model. For *Y*0 = 2, in the case of channels 4–6, the algorithm generated an error, as it had too few previous observations to correctly estimate the model; therefore, the value of *Y*0 was increased to 6 (Mdl.P + 5).

In order to assess the quality of model fitting, variance plots of the models were generated for individual channels, assuming ARIMA model parameters equal to one, as shown in Figure 12. Based on the illustrated figure, it can be stated that the variance level is channel-dependent and is in the range of 20 ÷ 35 for measurements form sensors closer to the middle notch of the specimen (ch1 and ch6). For the rest of channels, the variance is in the range of 15 ÷ 20, with the lowest values observed for channel 3.

Due to the observed high variance values for the obtained models, an automatic optimization of ARIMA model parameters was performed for two data series originating from different sensors locations. This process began with checking whether a single integration of the series, i.e., parameter *d* = 1, was sufficient to obtain a stationary series. The obtained partial autocorrelation functions indicate that a single integration is sufficient, as indicated by value close or even to 1 for lag 1. In Figure 13, which shows the partial autocorrelation function after single integration, a clear decrease in values for successive lags can be observed. It can be noted that for channel 4, slightly higher values are observed for the second and third lag compared to channel 1.

After verifying the stationarity condition of the series, the values of *p* and *q* were optimized for the two data series using the *aicbic* function. For the first dataset, these parameters were 5 and 4, respectively, and for the second dataset, 5 and 5. Due to the large amount of data to be analyzed and their high variability, the series were averaged, which reduced computation time and improved prediction accuracy, as shown in Figure 14 and Figure 15. The variance values were then recalculated, and for the determined models they were found to be significantly reduced by 3 orders of magnitude, compared to the preliminary model. That is a clear quantitative factor, which indicates a better fit of the models for a presented time series data.

Furthermore, prediction error values expressed as percentages were calculated for two datasets forecasted for channel 1 and channel 4, using parameter-optimized ARIMA models. The results of the analysis are shown in Figure 17 and Figure 18. The 10% error threshold is indicated by an orange line, whereas the 15% threshold is marked in red. The plots demonstrate that the majority of the generated models estimate the sensor’s output voltage with an error below 10%. For channel 1, prediction errors greater than 10% occur in 2.3% of the data, and errors exceeding 15% occur in 1% of the forecasted samples. For channel 4, these percentages are 6.4% and 4.1%, respectively.

## 5. Conclusions

The paper analyzes three basic groups of time series models: adaptive, analytical, and autoregressive models, and gives examples of other more advanced solutions. The dedicated data processing algorithm will be based on the adopted time series model, allowing for the prediction of its future values.

Taking into account the nature of the variables generated by the crack detection sensor, models characterized by a constant value of the variable, a trend, and a random component were analyzed in detail. Methods taking into account seasonality and giving the possibility of forecasting only one time step forward were rejected. For this reason, the ARIMA model, among autoregressive methods, was selected for preliminary testing of the data processing algorithm. It was also proposed that the data obtained by the data acquisition system could be divided into intervals, where other models would be used to take into account the different nature of the data.

In the approach presented in the paper, the technique is adopted to monitor fatigue cracks in metal structures (e.g., aircraft, infrastructure, automotive), as different types of fatigue failure characterize composites. Moreover, its future potential can be enhanced by targeting the work, e.g., to monitor the bond line edge of composite patch repair of the structure or to detect damages from the impacts of composite structures with an embedded sensing layer composed of a 2D conductive grid.

The focus of the work was to evaluate prediction feasibility under controlled model complexity, and ARIMA provided an interpretable baseline that could be consistently applied across all sensors. Future work will include a comparison with additional forecasting methods to further validate the findings.

## Figures and Tables

**Table 1:** Parameters of predictive algorithm.

Parameter Name	Label	Value
Order of the AR component	*p*	1
Order of the MA component	*q*	1
Degree of integration	*d*	1
Number of based samples(1 iteration)		20,000
Number of predicted samples (1 iteration)		1000
Number of measured samples		7,461,396
Presample response data (channel 1–3)	Y0	2
Presample response data (channel 4–6)	Y0	6

**Table 2:** Calculation time and number of models for every analyzed case.

Number of Case	Name of Channel	Calculation Time [s]	Number of Models
Case 1	MTC_ch1	1373.21	7400
Case 2	MTC_ch2	693.89	7400
Case 3	MTC_ch3	617.80	7440
Case 4	MTC_ch4	1068.55	7440
Case 5	MTC_ch5	1006.95	7440
Case 6	MTC_ch6	1224.45	7440

## Data Availability

The data presented in this study are available on reasonable request from the corresponding author due to legal reasons.

## Abbreviations

The following abbreviations are used in this manuscript:

CCPS	Customized Crack Propagation Sensor
ARIMA	Autoregressive Integrated Moving Average
SARIMA	Seasonal Autoregressive Integrated Moving Average
LSTM	Long Short Term Memory
ANN	Artificial Neural Network
VAR	Vector Auto Regression
MRMRMS	Minimum Redundancy Maximum Relevance and Maximum Synergy
SVR	Support Vector Regression
RF	Random Forest
